# Supplementary figures and images for: A Rare Case of Dedifferentiated Liposarcoma with Osteosarcomatous Differentiation-Diagnostic and Therapeutic Challenges
Source: Diseases. 2023 Dec 25;12(1):6. doi: 10.3390/diseases12010006 (PMC10814935; doi:10.3390/diseases12010006)

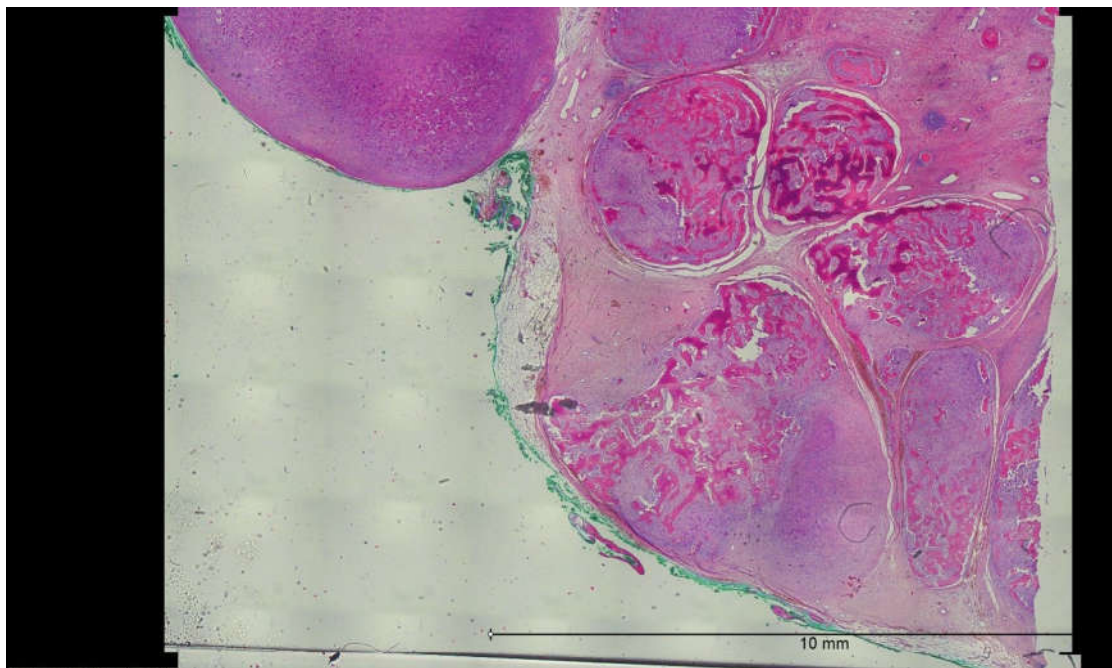

Figure S1. Capsule microphotograph

Supplement: Supplementary file 1 [file diseases-12-00006-s001.zip › diseases-2750177-supplementary.pdf]
